# Genomic architecture of FGFR2 fusions in cholangiocarcinoma and its implication for molecular testing

**DOI:** 10.1038/s41416-022-01908-1

**Published:** 2022-07-23

**Authors:** Olaf Neumann, Timothy C. Burn, Michael Allgäuer, Markus Ball, Martina Kirchner, Thomas Albrecht, Anna-Lena Volckmar, Susanne Beck, Volker Endris, Hannah Goldschmid, Ulrich Lehmann, Huriye Seker-Cin, Sebastian Uhrig, Stephanie Roessler, Jan Budczies, Stefan Fröhling, Thomas Longerich, Alex H. Wagner, Arndt Vogel, Peter Schirmacher, Albrecht Stenzinger, Daniel Kazdal

**Affiliations:** 1 Institute of Pathology, University Hospital Heidelberg, Heidelberg, Germany; 2 Center for Personalized Medicine Heidelberg (ZPM), Heidelberg, Germany; 3 German Cancer Consortium (DKTK), Heidelberg, Germany; 4 Formerly employed by Incyte Research Institute, Wilmington, DE USA; 5 Translational Lung Research Center (TLRC) Heidelberg, German Center for Lung Research (DZL), Heidelberg, Germany; 6 Institute of Pathology, Hannover Medical School, Hannover, Germany; 7 Computational Oncology Group, Molecular Precision Oncology Program, National Center for Tumor Diseases (NCT) Heidelberg, Heidelberg, Germany; 8 German Cancer Research Center (DKFZ), Heidelberg, Germany; 9 Department of Translational Medical Oncology, National Center for Tumor Diseases (NCT) Heidelberg, Heidelberg, Germany; 10 Liver Cancer Center Heidelberg, Heidelberg, Germany; 11 The Steve and Cindy Rasmussen Institute for Genomic Medicine, Nationwide Children’s Hospital, Columbus, OH USA; 12 Department of Pediatrics and Biomedical Informatics, The Ohio State University College of Medicine, Columbus, OH USA; 13 Gastroenterology, Hepatology, and Endocrinology, Hannover Medical School, Hannover, Germany; 14 Present Address: Tyra Biosciences, Carlsbad, CA USA

**Keywords:** Bile duct cancer, Predictive markers

## Abstract

**Background:**

Cholangiocarcinoma (CCA) is a primary malignancy of the biliary tract with a dismal prognosis. Recently, several actionable genetic aberrations were identified with significant enrichment in intrahepatic CCA, including *FGFR2* gene fusions with a prevalence of 10–15%. Recent clinical data demonstrate that these fusions are druggable in a second-line setting in advanced/metastatic disease and the efficacy in earlier lines of therapy is being evaluated in ongoing clinical trials. This scenario warrants standardised molecular profiling of these tumours.

**Methods:**

A detailed analysis of the original genetic data from the FIGHT-202 trial, on which the approval of Pemigatinib was based, was conducted.

**Results:**

Comparing different detection approaches and displaying representative cases, we described the genetic landscape and architecture of *FGFR2* fusions in iCCA and show biological and technical aspects to be considered for their detection. We elaborated parameters, including a suggestion for annotation, that should be stated in a molecular diagnostic *FGFR2* report to allow a complete understanding of the analysis performed and the information provided.

**Conclusion:**

This study provides a detailed presentation and dissection of the technical and biological aspects regarding *FGFR2* fusion detection, which aims to support molecular pathologists, pathologists and clinicians in diagnostics, reporting of the results and decision-making.

## Introduction

### Histological, molecular and therapeutic aspects of cholangiocarcinoma

Cholangiocarcinoma (CCA), representing the second most frequent type of primary liver cancer, is a rare cancer with an annual incidence ranging from 0.72 to 1.62 per 100,000 individuals in the United States [1]. CCA exhibits an aggressive course, which is further aggravated by late presentation in most patients, resulting in a dismal prognosis with a 5-year survival rate of 5–20% [2].

According to its anatomical location, CCA is categorised into intra- and extrahepatic cholangiocarcinoma (iCCA and eCCA) with the latter being further subdivided into perihilar (pCCA) and distal cholangiocarcinoma (dCCA). Though clinical management is still largely based on this classification scheme, recent studies provide accumulating evidence that for iCCA this classification is not sufficiently detailed [3–5]. Considering shared clinical, aetiological, histological and molecular features, as described by the new WHO classification of gastrointestinal tumours, iCCA is now further subclassified into a small- and large-duct type [6] (Suppl. 1). While large-duct type iCCA is often paralleled by the presence of biliary precursor lesions and mutations in *KRAS* (hgnc:6407) and *TP53* (hgnc:11998) similar to eCCA, genetic alterations in *IDH1/2* (hgnc:5382/hgnc:5383), *BAP1* (hgnc:950), *BRAF* (hgnc:1097) and *FGFR2* (hgnc:3689) are almost exclusively found in small-duct type iCCA [4, 7]. Given the potential druggability of the latter genetic events, this subdivision will gain increasing relevance with the rise of targeted molecular assays for the clinical management of unresectable cases.

Studies have shown that up to 20% of iCCA harbour *IDH1* and *IDH2* mutations [8, 9]. Activating mutations and gene fusions involving *FGFR2* were mutually exclusive with these and identified in 4% [10] and 10–15% [11, 12] of intrahepatic bile duct carcinomas, respectively. *FGFR2* alterations are restricted to the small-duct subtype of iCCA. This study focuses on *FGFR2* fusions that exhibit a diverse and steadily growing number of fusion partners and translocation breakpoints. Over the last few years, selective oral inhibitors targeting FGFR1/2/3 (hgnc:3688/3689/3690) were developed. A recent multicenter, open-label, single-arm, multicohort, Phase II study (FIGHT-202) [13], which investigated the FGFR2-Inhibitor Pemigatinib (INCB054828), reported an overall response rate (ORR) of 35.5% and a disease control rate (DCR) of 82% in 107 patients with *FGFR2* fusions or associated rearrangements. These data led to the approval of Pemigatinib as a second-line treatment for patients with metastatic/advanced CCA harbouring a *FGFR2* fusion or another *FGFR2*-associated rearrangement, first by the FDA and more recently by the EMA. Based on these encouraging data, an open-labelled, randomised, Phase III study (FIGHT-302) is underway to evaluate Pemigatinib as first-line treatment vs. conventional chemotherapy. Moreover, the FGFR inhibitors Erdafitinib and Rogaratinib were evaluated in CCA patients as part of an entity-independent basket trial and Erdafitinib was tested in a Phase II study of 14 patients with biliary tract cancers and FGFR alterations including activating mutations and fusions. The study demonstrated a disease control rate of 83.3% with a median progression-free survival of 5.6 months. Larger Phase II studies with Infigratinib (BGJ398) [14] and Futibatinib [15] showed an objective response rate of 23.1% and 25.4% for fusion-positive iCCA, respectively. Taken together, these data herald a paradigm shift in the clinical management of iCCA, which translates compellingly into comprehensive molecular profiling of these tumours to reflect the new standard of care. It will be of importance to establish reliable molecular pathological methods in routine diagnostics to be able to identify all treatable genetic alterations especially *FGFR2* gene fusions and rearrangements. Given the immense diversity of *FGFR2* fusions, both a detailed understanding of the genetic landscape of *FGFR2* aberrations and thorough knowledge about the technology and methodology employed for detection is crucial. Here we report the detailed analysis of the diverse genetic architecture of *FGFR2* gene fusions and derive implications for selection of available methods in molecular diagnostics as well as for the annotation in diagnostic reports. We put a specific focus on the advantages and disadvantages of widely-used, focused, next-generation sequencing-based methodologies, which will to our mind become the gold standard for gene fusion analysis in a routine clinical setting.

### Detailed consideration of FGFR2-associated rearrangements

#### FGFR2 Structure: exonic regions and protein domains

The fibroblast growth factor receptor 2 (*FGFR2*) is a member of a gene family of four receptor tyrosine kinases (*FGFR1-4*) that are involved in several crucial cell functions including angiogenesis, differentiation, development, survival, tissue repair, and proliferation [16]. The gene locus of *FGFR2* is located on the reverse strand of the long arm of chromosome 10 (10q26.13) spanning nucleotide positions 123,237,844 to 123,357,972 (hg19, alignment from ncbigene:2263). At the DNA level, at least 24 (according to NCBI; www.ncbi.nlm.nih.gov/gene/2263) regions exist that qualify as exonic sequences, but only subsets of these are used for different isoforms through alternative splicing, resulting in a vast variability on transcript level. To the present day, more than 25 isoforms of *FGFR2* have been described.

**Fig. 1:**
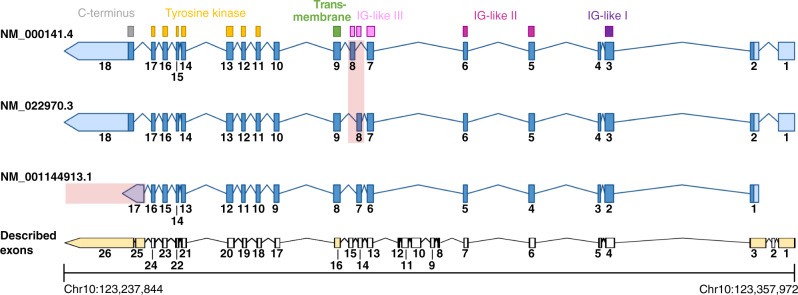
Schematic representation of selected FGFR2 isoforms. Shown is the genetic structure of the two principal isoforms NM_000141.4 = FGFR2 IIIc and NM_022970.3 = FGFR2 IIIb expressed by mesenchymal and epithelial cells, respectively. Both isoforms differ only by the use of an alternative eighth exon (highlighted in red) that alters a part of the third immunoglobulin (Ig)-like domain. The isoform NM_001144913.1 represents a variant of the FGFR2 IIIb isoform lacking the first exon and using a shorter alternative last exon which leads to a shortened C-terminus also known as C3 terminus (highlighted in red). Blue bars and numbering on each isoform depict the encoded exon, where dark blue indicates coding sequences and light blue untranslated regions. At the bottom, a representation of 26 described exonic regions is displayed in black. Exons highlighted in yellow indicate that alternative exon regions exist at this genomic position. The lines connecting the exons represent the corresponding intronic regions but scaled down to 15% of the original length for illustrative purposes.

The canonical isoform of *FGFR2* refers to the transcript NM_000141.4 (also named FGFR2 IIIc), consisting of 18 exons which code for an 821 amino acids (aa) long protein that is expressed in mesenchymal cells. It differs from the transcript NM_022970.3 (FGFR2 IIIb), which is predominantly expressed by epithelial cells, only by an alternative eighth exon that alters a part of the third immunoglobulin (Ig)-like domain and results in elongation of the protein by one amino acid to a total of 822 aa. Both isoforms share three extracellular Ig-like domains (Ig-like I, aa 43–115; Ig-like II, aa 172–248; Ig-like III, aa 264–359), a heparin-binding domain (aa 162–178), a transmembrane domain (aa 371–401), a tyrosine kinase domain (aa 481–757), and a proline-rich C-terminus containing multiple tyrosine phosphorylation sites, but differ in their binding affinity for different fibroblast growth factors (FGF). Of note, for mutation and annotation purposes, the canonical isoform NM_000141.4 is mostly used, although from a biological point of view, the isoform NM_022970.3 is expected to be expressed in epithelial tumours, such as iCCA. This issue can be seen as a purely formal aspect since oncogenic alterations within the alternative eighth exon are rare and oncogenic translocations do not occur there. As long as the RefSeq ID is specified with the detected alteration, the annotation is comprehensible and can be converted to another isoform if necessary. A schematic graphical representation of these two isoforms is shown in Fig. 1. In addition, the isoform NM_001144913 which encodes the oncogenic FGFR2 IIIb C3 is provided.

Under wild-type conditions, simultaneous binding of an FGF and heparin/heparan sulfates as co-factors to FGFR2 leads to conformational changes that induce dimerisation and subsequently trans-autophosphorylation of the receptor at its cytoplasmic part. The activated intracellular kinase domain is now able to phosphorylate downstream targets such as FRS2, leading to the activation of various signalling pathways, including MAPK, PI3K-AKT, and JAK-STAT.

#### Oncogenic activation of FGFR2

The ligand-independent activation of FGFR2 is a known oncogenic event [17] predominantly found in intrahepatic cholangiocarcinoma with a prevalence of around 13% [11, 18]. But it is also present at low frequencies in many other tumour entities including but not limited to breast, endometrial, gastric, lung, or thyroid cancer. Two main mechanisms are described for the ligand-independent activation of FGFR2 as a result of a genetic rearrangement, which will be discussed below: (I) the loss of the C-terminal region of FGFR2 and (II) the gain of domains enhancing dimerisation.

##### C-terminal truncation

Alternative splicing of *FGFR2* can also affect the C-terminal region and result in shortened proteins, described as C2 and C3 variants [19], lacking the last 34 or 53 aa, respectively. Breast cancer cell lines expressing an isoform with a C3 terminus (e.g. NM_001144913.1 or NM_001144919.2) show potent transformation capability and ligand-independent, constitutive FGFR2 activation, indicated by strong phosphorylation of FRS2 (hgnc:16971) [20]. Similar results showing an enhanced transforming activity of the C3 variant are described in other studies [21]. The oncogenic effect of these C-terminal deletions, which may encompass the entire last exon (exon18, NM_000141.4), is attributed to aberrant receptor internalisation and enhanced FRS2-dependent signalling, caused specifically by the loss of the YLDL motif [22] (aa 769–772, NM_000141.4). The proline-rich C-terminal region acts as a binding site for the SH3 domain of the growth factor receptor-bound protein 2 (GRB2, hgnc:4566). GRB2 is a critical component in the downstream signalling of almost all receptor tyrosine kinases. But it was also shown to have a role in the negative regulation of FGFR2 by sterically hindering C-terminal phosphorylation and adapter protein recruitment [23]. Truncation of the last 10 aa of FGFR2 can impair the binding of GRB2 and their interaction [24]. Thus C-terminal truncation, which can be introduced by a deletion, by a splice site mutation, or by a translocation, represents an independent mechanism of FGFR2 activation. For fusions that can be assigned to this mechanism, it does not matter whether functional domains are obtained from the fusion partner, or whether the reading frame is retained within the fusion product.

##### Enhanced dimerisation

As mentioned above, binding of FGF leads to FGFR2 activation by inducing receptor dimerisation. A gene fusion leading to additional dimerisation or oligomerization domains may enhance the dimerisation ability of FGFR2, or may even make the fusion protein independent of FGF binding [11, 25]. This is observed in the vast majority of *FGFR2* fusions with intact reading frames. Here, *FGFR2* acts as 5’ fusion partner, with breakpoints occurring downstream of the intracellular tyrosine kinase domain most frequently but not limited to within intron 17 (NM_000141.4). A multitude of different 3’ fusion partners was described [26]. The common denominator of these is, that the sequence contributed to the fusion contains the code for strong dimerisation/oligomerization domains, like AFF3, BAG, BAR, coiled-coil, FN1, leucine zipper, LIS1, SAM, SPHF, or zinc finger [25, 27].

To accurately identify fusions that use this mechanism, it is imperative to identify the precise fusion junction in order to determine which protein domains are intact and functionally conserved in the fusion protein.

## Materials and methods

A detailed genetic analysis of the original data from the FIGHT-202 trial (NCT02924376) [13], including DNA-seq [28] and RNA-seq [29] data was conducted using the R software (v.4.0.2; R Core Team, 2016).

For visualisation of genomic regions, the IGV browser (https://igv.org/) [30] and the UCSC genome browser (http://genome.ucsc.edu) [31] were used. For a schematic representation of gene fusion architectures, ARRIBA [32] was used.

## Implications for testing and reporting

### Test strategies

#### DNA- vs RNA-based NGS: sequencing capabilities, breakpoint vs fusion junction, transcription level, isoforms

**Fig. 2:**
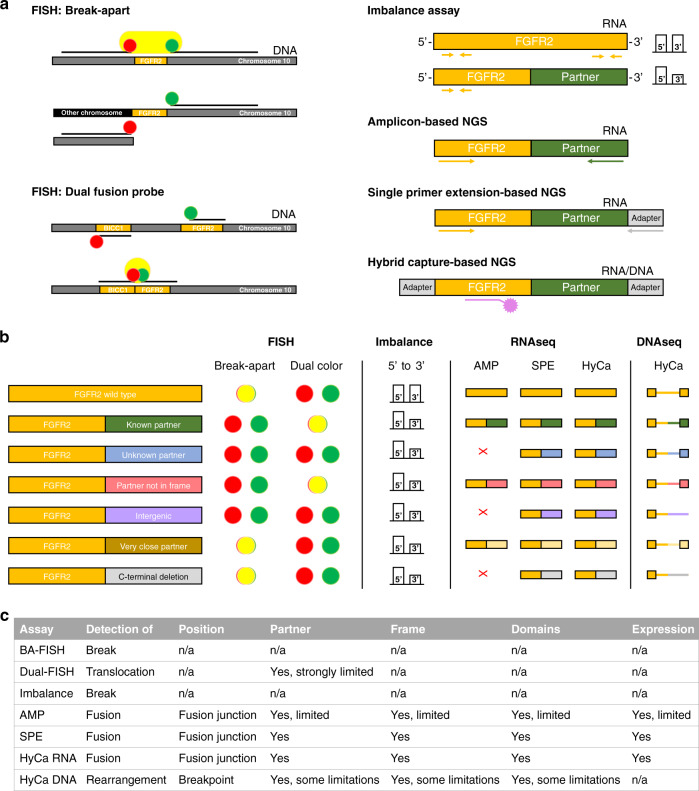
Comparison of different methods for the detection of *FGFR2* translocations/fusions. **a** Main principle of fluorescence in situ hybridisation using break-apart probes (BA) and dual fusion probes (Dual), imbalance assay, amplicon (AMP)-based, single-primer extension (SPE)-based, and hybrid-capture (HyCa)-based NGS. **b** Theoretical performance of the different assays for specific fusion events. The first two rows provide the results for the wild-type and a known fusion partner as references. FISH: Separate or overlapping fluorescence signals are indicated by green and red dots or a yellow dot, respectively. Imbalance: The bars represent the number of RNA molecules detected considering the 5’ or 3’ region. RNA-seq and DNA-seq: Red cross indicates that a fusion event was not detectable by an assay. Wide and narrow bars represent exonic and intronic regions, respectively. **c** Characteristics and informative value of the different assays.

Detection of *FGFR2* rearrangements and fusions with DNA- and RNA-based approaches are routinely utilised by clinical labs. However, key differences in technologies must be considered when interpreting results. The main principles of different methods for the detection of *FGFR2* fusions/translocations on DNA- or RNA level are depicted in Fig. 2a. The break-apart FISH (BA) approach uses two probes near the *FGFR2* locus that result in a combined fluorescence signal (yellow) for the wild-type situation, which is lost if a DNA break occurs between the two probes, resulting in separate fluorescence signals for each probe (green and red). The principle is reversed for the dual fusion probe (Dual). Here, two gene-specific probes are used rendering separate fluorescence signals (red and green) for the wild-type situation. In the event that the two probed loci are fused, the fluorescence signals overlap and result in a yellow fusion signal. While BA-FISH assays detect rearrangements in a partner-agnostic manner, they lack the granularity of sequencing-based methods and provide no information on gene partners. Dual-FISH assays on the other hand can identify one specific fusion partner, but their detection spectrum is limited to fusions involving this exact partner. Both FISH approaches cannot assess the expression or structure of a fusion protein.

A fusion partner-agnostic approach on RNA level that can be used to identify likely rearrangement events is the so-called imbalance assay. Here, the relative abundance of RNA molecules is determined by considering the 5’ and 3’ region of the *FGFR2* mRNA separately and looking for an imbalance between the two ends of the transcript. Wild-type *FGFR2* would result in similar counts for the 5’ and 3’ region, while a translocation involving the 3’ region would lead to a decrease of respective counts.

Regarding different NGS approaches one can differentiate between “closed” and “open” assays. Amplicon-based approaches are considered closed because they can only detect gene fusions for which a corresponding primer pair is included in the panel design. The single-primer extension approach (SPE; e.g., multiplexed anchored PCR) is an “open” assay capable of detecting gene fusions regardless of the fusion partner. This is achieved by relying only on one gene-specific primer. The primer-binding site used for the fusion partner is introduced through ligation of an adaptor. For hybrid-capture (HyCa)-based assays, target enrichment before sequencing is performed by using sequence-specific hybridisation probes. Therefore, the targeted RNA molecules are captured regardless of whether the sequence continues as wild-type *FGFR2* or with an alternative sequence as a result of a fusion event.

Applying the different assays can result in different and sometimes discordant results. Figure 2b displays exemplary results than can be expected for specific translocation events. Comparing the results for an unknown fusion partner, an out of frame fusion, an intergenic fusion, a very close fusion partner regarding the chromosomal position, or just a deletion of the 3’ region, it becomes evident that the results of different assays provide a variable depth of detail, which can cause misinterpretation. For example, both FISH approaches and the imbalance assay are not capable of detecting whether a fusion partner is in-frame. Furthermore, 3’ deletions of *FGFR2* cannot be detected by FISH and false-negative results can be expected for fusion partners that are in close proximity to *FGFR2*. While the imbalance assay would be sufficient to detect all displayed examples, it cannot differentiate between them. In regards to NGS approaches, all examples would be detectable using SPE- or HyCa-based assays (DNA & RNA), with the added benefit that the biological underpinnings of each exemplary fusion could be identified. But the amplicon-based approach would fail to detect fusions with an unknown or intragenic partner and, if not specifically designed for it, possible 3’ deletions of *FGFR2*. A summary of the details that can be gained with each assay is given in Fig. 2c.

When deciding which NGS panel to use for *FGFR2* fusion detection, there are different considerations to make. For DNA-based assays, the actual DNA breakpoint must be sequenced, requiring tiled hybrid-capture probes across the chromosomal breakpoints, which in the case of *FGFR2* have to involve exon17, intron 17, and exon18, all downstream of the kinase domain. Chimeric sequence reads with *FGFR2* and non-contiguous genomic sequences are identified and clustered to define the breakpoint region with the gene partner being computationally inferred based on the alignment of the non-*FGFR2* sequences to the genome. As an advantage, this approach is partner gene agnostic and only relies on the alignment of the chimeric sequence cluster to the reference genome to identify partner genes. Given the computational inference, cases exist that exhibit a breakpoint in the partner gene which is not in-frame or on-strand. In these cases where a fusion partner cannot be definitively identified, the alteration is reported as a rearrangement rather than a fusion. In addition, fusions with breakpoints in the intergenic space will be reported as rearrangement with no partner gene. Published reports for *FGFR2*-rearranged cholangiocarcinoma indicate that non-partner rearrangements are reported in ~10% of all cases [28, 33]. These alterations result from a variety of molecular events including bridged fusion where non-contiguous DNA is inserted in the breakpoint between *FGFR2* and the partner gene, or true intergenic rearrangements that provide a downstream cryptic C-terminal exon. In clinical trials so far no obvious differences in response rates were reported for *FGFR2* fusions or *FGFR2* rearrangements without a partner gene identified [28]. However, further analyses are needed to investigate potential prognostic and/or predictive effects of different fusion partners, particularly with regard to the introduction of oligomerization domains or the development of resistance mechanisms.

In contrast, RNA-based methodologies for *FGFR2* fusion detection interrogate the actual fusion transcript. As such, no computational inference is needed as the exons at the fusion junction and the conservation of the reading frame are directly assessed at the level of the primary sequence, making the reliable identification of the fusion partner and the affected exons more robust and straightforward in practice. Especially for the latter, DNA-based approaches were described as an unreliable predictor [34]. But there are key assay features for evaluating an RNA-based *FGFR2* fusion detection. Most importantly the assay must be fusion partner-agnostic and should not rely on gene-specific primer pairs. This implies that amplicon-based panels are not suitable for this purpose, because they are limited to a predefined set of fusions determined by the primer design. This is best illustrated by the fact that more than 140 partner genes were so far identified for *FGFR2* fusions in CCA [33]. RNA-based, partner-agnostic assays can be based on standard RNA-seq, single-primer extension, or 5’/3’ imbalance assays looking at the discordance between transcript counts for the 5’ and 3’ ends of the *FGFR2* mRNA. By analysing information at the transcription level, RNA-based assays provide a proof of the first step of fusion gene expression. Transcription also confers greater sensitivity to RNA-based assays compared with DNA-based assays, as it leads to a signal enhancement due to larger numbers of RNA molecules compared to DNA molecules. A disadvantage of RNA-based analyses is the instability of RNA that may especially become evident with routinely used formalin-fixed paraffin-embedded (FFPE) tissues and associated processing and physical storage conditions, that have a great impact on RNA preservation and more importantly RNA degradation.

Another important factor regarding the appropriate *FGFR2* fusion detection method is the amount of tumour cells and thereby tumour DNA/RNA in the sample. Sufficient tumour DNA/RNA is crucial for a valid identification of the alterations and to prevent false-negative results, e.g. due to low tumour cell content. An estimation of different detection limits and quality parameters is published for various assays [35, 36]. According to our (single centre) experience, small biopsies of cholangiocarcinoma are well suited for combinatorial DNA and RNA profiling by HyCa or SPE-based assays with a dropout rate of <8%. A partner-agnostic NGS approach is the best choice for detecting *FGFR2* fusions, since partner-specific approaches will perform poorly due to the already discussed pronounced heterogeneity of fusion partners. Ideally, a combined RNA-based fusion and DNA-based translocation detection is applied. While this approach would comprehensively investigate fusion events, it may not be sufficiently efficient in terms of time, cost, and tissue consumption in a diagnostic setting. DNA translocation analysis can be pursued alternatively, if the hotspot translocation areas are sufficiently covered by primers/probes. If a sample is not sufficient for NGS-based analysis, a *FGFR2* break-apart FISH should be used for analysis, since this analysis needs only 50–100 cells for reliable detection of a possible *FGFR2* translocation. A decision tree for the selection of the appropriate technology for the detection of *FGFR2* translocations/fusions is given in Suppl. 2.

For DNA- and RNA-based assays the location of the breakpoint or fusion junction is important. Exon17 of *FGFR2* encodes the C-terminal end of the kinase domain; thus, only rearrangements involving exon17, intron 17, or the protein-coding region of exon18 will maintain the kinase domain and can act oncogenic. Given that all *FGFR2* fusions in iCCA are “type 2” fusions with C-terminal fusion partners, any rearrangements upstream of exon17 will not contain a functional kinase domain and would be expected to be insensitive to an FGFR inhibitor. As such, diagnostic reporting should reflect this consideration. A general restriction of DNA and RNA-based NGS assays is the maximum read length with high data quality between ~100 bp and 250 bp, depending on the sequencing technology. Since RNA or DNA extracted from FFPE material is degraded, the short read length is not a compromise but in fact well suited for sequencing in a routine molecular pathology setting. Sequence details covering the breakpoint or fusion junction can be obtained with a certain amount of sequence information from both partners, but this approach cannot provide information about a full-length fusion transcript.

#### Implications for DNA-seq-based *FGFR2* translocation analysis

**Fig. 3:**
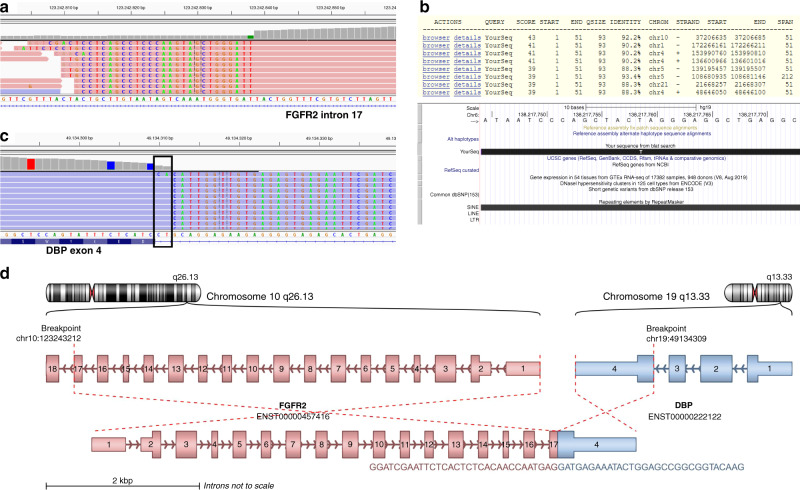
Example of a *FGFR2* fusion where the fusion partner could not be identified by DNA sequencing. **a** The identified translocation point localised in intron 17 of *FGFR2* (NM_000141), shown in the IGV browser (30). The aligned soft-clipped sequences are part of an Alu repeat. **b** Upper part: Part of a BLAT result list (>100 results) of a representative split read from (**a**). Lower part: Expanded view of one of the results showing the split read sequence in reverse complement using the UCSC genome browser (31). It is part of a SINE repeat (AluSx family). **c** RNA-seq identifies the fusion partner as DBP (hgnc:2697). Displayed is the fusion junction in DBP with soft-clipped reads that align to FGFR2, shown in the IGV browser (30). Of note, the two marked bases belong to the FGFR2 part (exon17) of the fusion transcript, but as they can be aligned to either fusion partner they were displayed by the IGV browser as aligned to the last two bases of DBP intron 3. **d** The putative full-length transcript of the fusion; rendered with ARRIBA (32).

A direct comparison of data from samples assayed by both DNA-based NGS and RNA-seq highlights the differences between the two assay formats and their impact on fusion reporting. RNA-seq analysis was performed on two samples from the Phase 2 FIGHT-202 study (NCT02924376), which had been reported as rearrangements with no identifiable gene partner by a DNA-based NGS (Foundation Medicine). In both cases, RNA-seq revealed in-frame *FGFR2::BICC1* (hgnc:19351) fusions. Based on the RNA and DNA analysis it is clear that in these cases intergenic sequences from chromosome 1 and chromosome 10, respectively, were incorporated into the genomic breakpoint generating “bridged” fusions [29] (Fig. 3). *FGFR2* intron 17 needs to be covered in its entire length by a sufficient amount of capture probes to enable the detection of intronic breakpoints. The example shows that cases exist in which only the breakpoint in *FGFR2* could be detected by the DNA-based approach, but the complete translocation event could not be resolved, because the complex recombination involved more than two chromosome arms/areas. In these cases, RNA analysis allows direct detection of the fusion event and provides information on whether the fusion is (1) in-frame, (2) contains intact structures like kinase domains that are important for therapy decision-making and (3) is expressed at all. Examples like this highlight the importance of reporting rearrangements involving the *FGFR2* hotspot region with no definitive partner gene identified by a DNA-based assay.

#### Implications for FISH-based *FGFR2* breakage/translocation detection

**Fig. 4:**
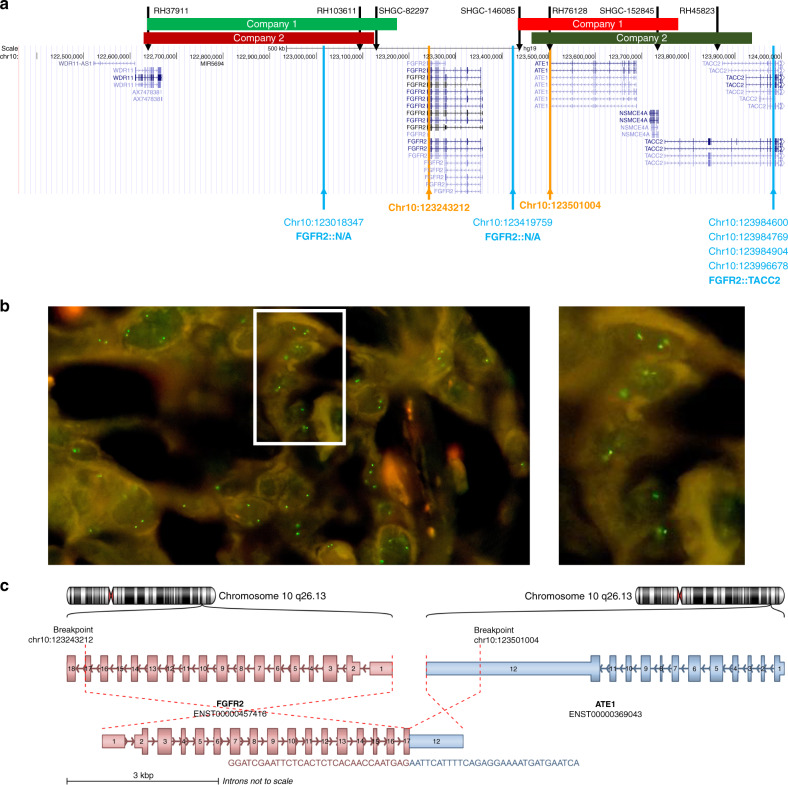
Break-apart FISH analysis of a FGFR2::ATE1 (F17A12) fusion with breakpoints after FGFR2 exon17 and in ATE1 exon12. **a** Representation of the chromosomal region of FGFR2 and ATE1 using the UCSC genome browser (31). The approximate hybridisation location of the FISH probes of 2 different assays are displayed as green and red bars (light colours company 1, dark colours company 2). Orange lines show the estimated breakpoints for FGFR2 and ATE1. The black arrows indicate STS markers used for mapping of the BAC clone positions. The blue arrows indicate breakpoints adjacent to FGFR2 identified in the FIGHT-202 study. **b** Representative FISH image of the FGFR2::ATE1 fusion-positive case. **c** Schematic representation of the putative gene fusion; rendered with ARRIBA (32).

FISH-based detection of chromosomal rearrangements is a well-established molecular diagnostic technology that is also fast, reliable, and cost-efficient. Two FISH approaches widely in use are the break-apart probes (BA) and fusion-specific probes (Dual) (Fig. 2). While the first approach is mostly fusion partner-agnostic, the second is specific and limited to one exact partner gene. Additionally, specific fusion variants or the expression of a putative fusion cannot be identified with this technique. The break-apart approach relies on the strategy that after a chromosomal rearrangement event the two probes are separated far enough from each other in order to assure visibility of the two different colour signals. Therefore, genomic rearrangements that do not sufficiently separate the two probes, as it is the case for genes that are in close proximity to each other, can yield false-negative results applying FISH analyses. In fact, this situation was shown for certain *FGFR2* rearrangements. As demonstrated in Fig. 4, a rearrangement of the *FGFR2* locus with the *ATE1* gene (hgnc:782), which is located in close proximity to *FGFR2*, cannot be detected with two commercially available probes. Similar cases, that might have been missed using FISH, were seen in 4,2% (four *TACC2* (hgnc: 11523) translocations and two intergenic translocations events in close proximity to *FGFR2*) of fusions identified in the cohort of the FIGHT-202 study.

#### Implications for RNA-seq *FGFR2* fusion analysis

RNA-based NGS approaches are the preferred method for the detection of oncogenic fusions. Its advantages are (I) the direct demonstration that a fusion is transcribed, indicating expression, (II) the possibility to determine if the fusion is in-frame and (III) the identification of the fusion partner and the involved exons. Practical limitations are the higher costs and the need to extract RNA from the sample. Three main techniques are currently used for the detection of *FGFR2* fusions, of which the HyCa- and SPE-based approaches are the most reliable, whereas the AMP-based technique is not preferable in this regard, given the large number and diversity of fusion partners (see above). Hybrid-capture probes cover all possible areas of the *FGFR2* gene locus that qualify as exons (Fig. 2), while SPE-based panels are designed for the canonical *FGFR2* isoform (NM_000141.4). Table 1A gives an overview of the exonic regions covered by different panels, representative for each of the three methods. With the given translocation breakpoints of the cohort, we calculated the putative fusions that most likely will arise and summarised our findings in Table 1B and C. The SPE-based technology is able to detect up to 94–97% of the possible rearrangements/fusions, depending on the panel. The HyCa-based panels can detect all of these fusions, as they include probes targeting all regions of interest. The predicted fusion calls for the amplicon-based OCAv3-RNA Panel show that with this panel only 28.5% of the putative fusions could be detected. This is based on the fact, that for AMP-based assays to be able to detect a certain *FGFR2* fusion, a primer for the specific exon of the fusion partner involved needs to be included in the panel design. The number of all relevant exons of all possible *FGFR2* fusion partners is simply too large to be accommodated in a single AMP panel. Therefore, trade-offs must be made that may result in shortcomings in certain cases. An example for this with respect to the fusion partners detected in the cohort is *CCDC6* (hgnc:18782). While specific primers for exons 1 and 2 are included in the panel, two of the four *FGFR2*::*CCDC6* fusions queried had breakpoints in exons 3 or 4 of *CCDC6* and thus could not be detected. Closed amplicon-based assays are clearly limited in detecting of oncogenic fusions with many different partners.

**Table 1:** Overview of the covered FGFR2 exons by different RNA-based.

The overall number of detectable fusions is shown in bold type. NGS.

**Fig. 5:**
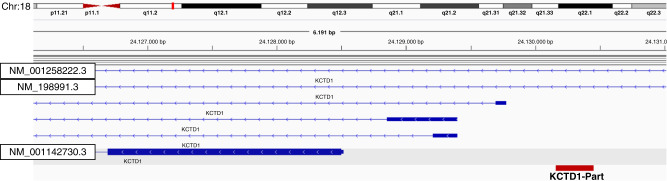
Fusion annotation considering alternative isoformspecific transcription start sites. Schematic representation of the KCTD1 breakpoint of a FGFR2::KCTD1 fusion in relation to the different isoforms encoded by this gene locus, shown in the IGV browser (30). Red bar: sequence of the fusion read aligned to KCTD1.

The following example shows issues that may arise during annotation, especially for fusions detected on DNA level. Figure 5 displays the case of a *FGFR2* fusion with the sequence of the fusion partner aligned to *KCTD1* (hgnc:18249). As depicted, different isoforms exist for *KCTD1* with different start codons, with the isoform NM_001142730.3 representing the canonical one. In relation to the canonical isoform this fusion is not in-frame, because the translocation point lies upstream of the start codon, which classifies the *FGFR2* fusion as a C-terminal truncation (compare 1.2.2.1). But when considering other *KCTD1* isoforms (NM_001258222.3 or NM_198991.3) the breakpoint would be in intron 1 resulting in an in-frame fusion, which might lead to a fusion with enhanced dimerisation capabilities (compare 1.2.2.2). Since no RNA-based analysis was performed here, it is not possible to determine which of these hypotheses applies.

## Parameters to include in a clinical translocation/fusion report

Considering the technical and biological aspects of fusion analysis discussed above, we recommend to include the following information in a molecular diagnostic report concerning *FGFR2* fusions. The goal should be to provide the clinician/oncologist with a brief but concise overview of how the analysis was performed and which information is given. This includes a short description of the assays used to isolate and prepare the sample, input quantity (RNA/DNA), and the fusion detection method applied. Quality parameters like tumour cell content and specific parameters concerning the quality of the nucleic acids as well as the prepared libraries are mandatory.

Up to date, there is no commonly accepted nomenclature for the diagnostic annotation of fusions and/or translocations. This is partly due to the different scientific communities involved. A technical annotation like the HGVS style complies with a short exact description, but it is hardly suitable for human reading. We recommend using the consensus nomenclature under development [37] by the Variant Interpretation for Cancer Consortium (VICC), which captures information regarding the transcript, associated transcript exons, and the offset of the exon boundaries from the fusion junction. A first public draft of these guidelines is expected to be released in 2022.

For a diagnostic report of *FGFR2* fusions detected by RNA sequencing, we strongly recommend to include the following information (see Suppl. 3 for an example):

- The number of reads (split/paired).
- The ratio of fusion reads, as the abundance of fusions transcripts compared to wild-type transcripts.
- The gene names of both fusion partners with according transcript IDs.
- The exons involved in the fusion junction.
- The information if the fusion is in-frame and if domains crucial for an oncogenic activity are intact.

For the annotation of translocations, we endorse following the recommendations of the International System for Human Cytogenomic Nomenclature (ISCN) and the Human Genome Variation Society (HGVS) [38]. Clinical reports of translocations detected by DNA sequencing should include (see Suppl. 4 for an example):

- The number of reads (split/paired).
- The ratio of reads showing the translocation.
- The names of the involved genes.
- The chromosomal position of the identified breakpoints.
- The human genome reference build used.
- Estimations regarding the preservation of the reading frame and protein domains, should be formulated cautiously.

## Conclusion

With the recent advancement in therapeutically targeting of *FGFR2* fusions in iCCA, standardised molecular profiling of these tumours will be necessary. Here we provide a comprehensive overview of the architecture of *FGFR2* rearrangements and discuss the implications for their reliable detection and interpretation. Comparing different approaches and based on representative cases from the FIGHT-202 trial we highlight biological and technical implications to be considered for analysis. Our data indicate that an appropriate NGS approach is recommended to reliably identify *FGFR2* gene fusion in cholangiocarcinoma. Finally, we point out parameters that should be included in a molecular diagnostic *FGFR2* report to enable full comprehension of the performed analysis and of the information provided about a detected translocation/fusion. Covering and dissecting these aspects in detail will provide a powerful resource for molecular pathologists, pathologists, and clinicians facilitating diagnostics, reporting of results as well as clinical decision-making.

## Supplementary information

Histopathology of small- and large-duct type intrahepatic cholangiocarcinoma.

Decision tree for FGFR2 detection assay selection

RNA-Report

DNA-Report

Academic Journals Reporting Checklist

## Data Availability

Not applicable.
