# Supplementary material for: Genomic architecture of FGFR2 fusions in cholangiocarcinoma and its implication for molecular testing
Source: Br J Cancer. 2022 Jul 23;127(8):1540–9. doi: 10.1038/s41416-022-01908-1 (PMC9553883; doi:10.1038/s41416-022-01908-1)
Supplement: Supplementary file 1 — Histopathology of small- and large-duct type intrahepatic cholangiocarcinoma. [file 41416_2022_1908_MOESM1_ESM.pdf]

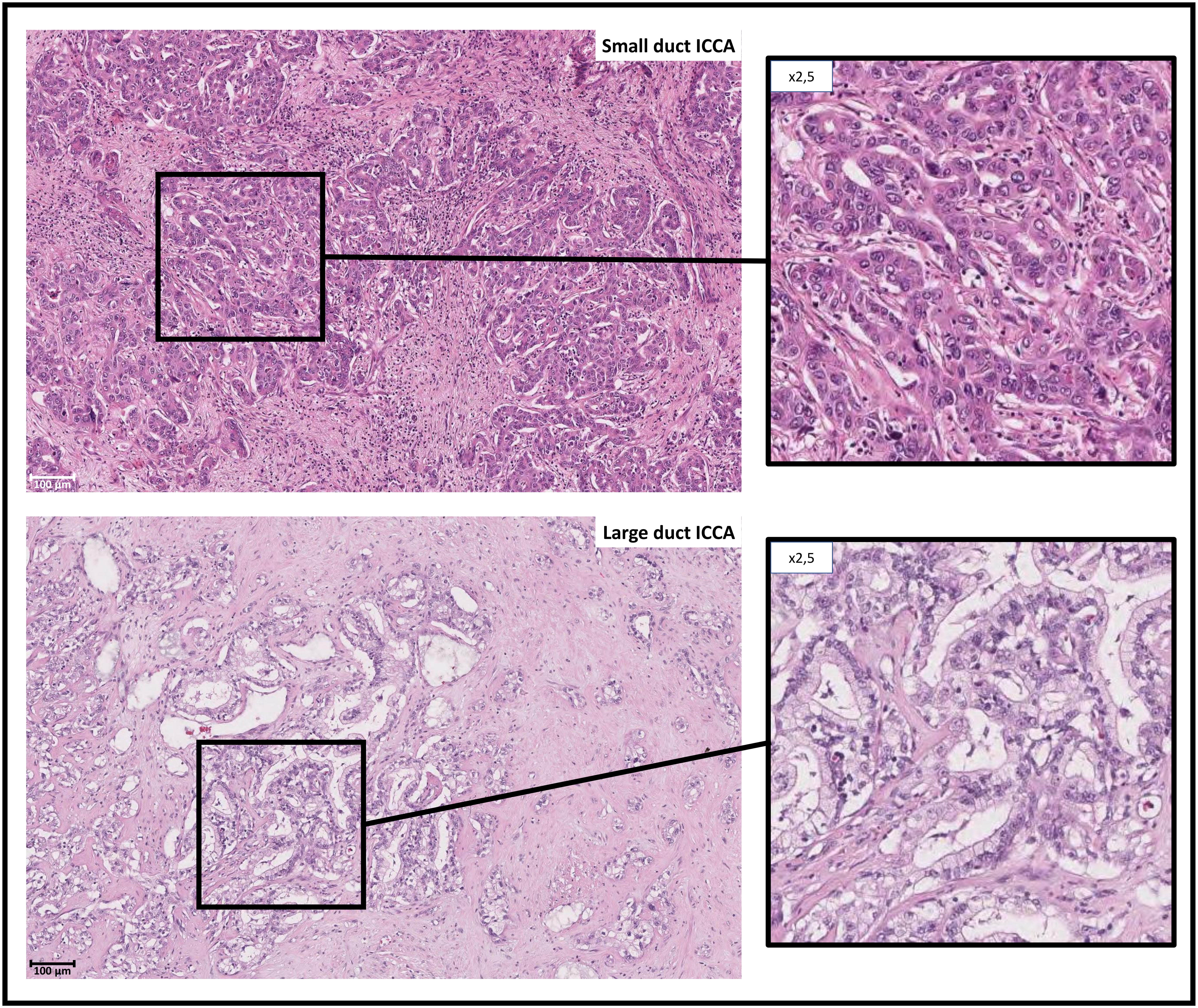

Suppl. 1: Histopathology of small- and large-duct type intrahepatic cholangiocarcinoma. Small-duct type intrahepatic cholangiocarcinoma upper panel) is characterized by cuboidal tumor cells forming small tubular or anastomosing glands with irregular lumina and absent mucin. In contrast, large-duct type intrahepatic cholangiocarcinoma (lower panel) is typically composed of tall columnar tumor cells with clear cytoplasm arranged in various-sized glandular structures and often associated with the presence of intra- or extracytoplasmic mucin. Original magnification 100X.
