# Supplementary figures and images for: Genomic architecture of FGFR2 fusions in cholangiocarcinoma and its implication for molecular testing
Source: Br J Cancer. 2022 Jul 23;127(8):1540–9. doi: 10.1038/s41416-022-01908-1 (PMC9553883; doi:10.1038/s41416-022-01908-1)

Suppl. 2: Decision tree for *FGFR2*  
detection assay selection

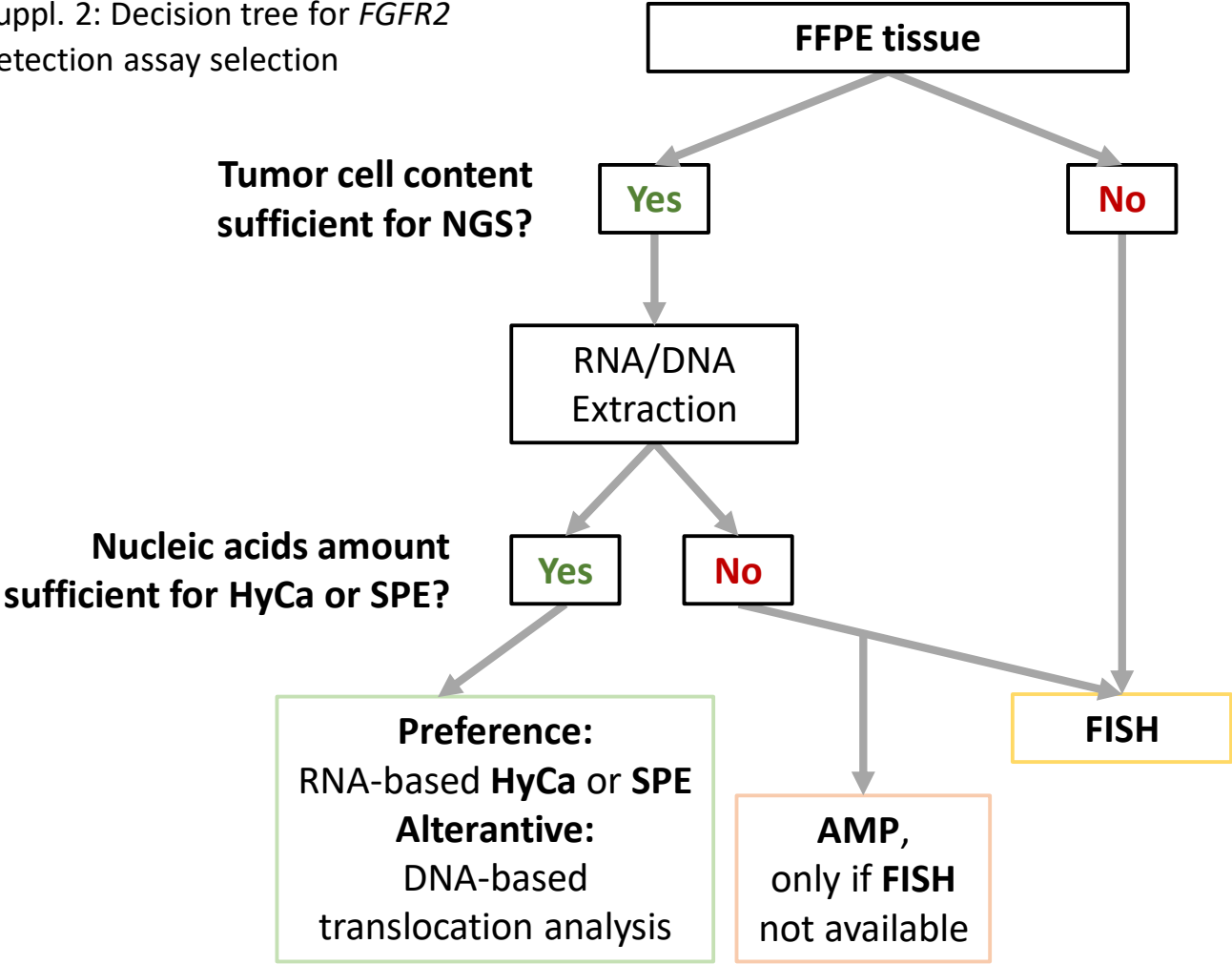

Supplement: Supplementary file 2 — Decision tree for FGFR2 detection assay selection [file 41416_2022_1908_MOESM2_ESM.pdf]
