# Supplementary material for: Genomic architecture of FGFR2 fusions in cholangiocarcinoma and its implication for molecular testing
Source: Br J Cancer. 2022 Jul 23;127(8):1540–9. doi: 10.1038/s41416-022-01908-1 (PMC9553883; doi:10.1038/s41416-022-01908-1)
Supplement: Supplementary file 3 — RNA-Report [file 41416_2022_1908_MOESM3_ESM.pdf]

**Material:**	Block 12345/6789
**Clinical Details:**	Request for RNA based NGS FGFR2 fusion analysis
**Histopathology:**	Cholangiocarcinoma

Report:

Identified Fusion

5' Fusion Partner	3' Fusion Partner	Fusion Supporting Reads	Ratio Fusion Reads
FGFR2 exon 17<br>(NM_000141.4)<br>*Fusion junction:* chr10:123243212	**BICC1 exon 3**<br>(NM_001080512.1)<br>Fusion junction: chr10:60461834	**4321**	**49 %**

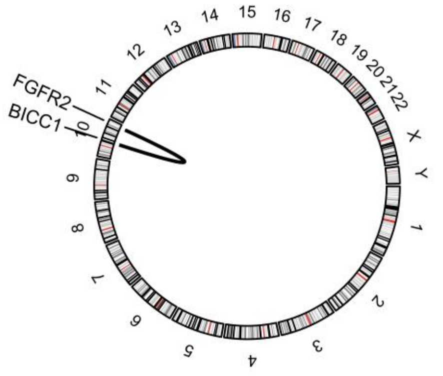

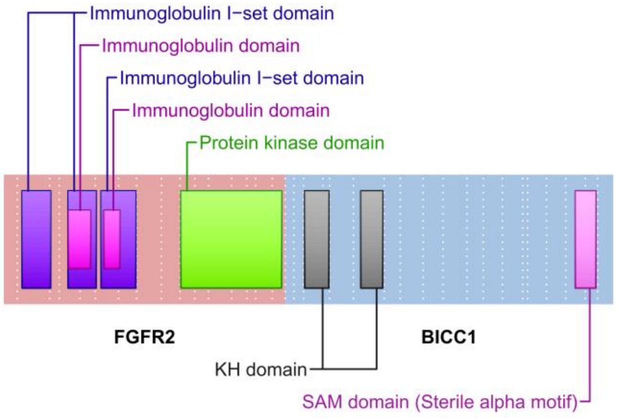

Comments – Interpretation:

In the present material an FGFR2::BICC1 fusion, involving FGFR2 exon 17 and BICC1 exon 3, was detected on RNA level. The fusion transcript results in the loss of the negative regulatory C-terminal region of FGFR2, while the kinase domain remains complete and intact. Further, a respective fusion protein would contain domains facilitating polymerization (KH and SAM) contributed by BICC1. The scientific literature describes both aspects as recurrent oncogenic mechanisms in cholangiocarcinoma (PMID: ######).

Additional information:

Pre-analytics

RNA input quantity:	200 ng	*Mapped Fragments:*	2000000
Tumor cell content:	70 %	*Unique mapped Fragments:*	999999

Performed analytics

Fusion analysis was performed on the morphologically confirmed and enriched tumor tissue (70% tumor cell content) using next-generation sequencing technology (SEQUENCING SYSTEM) using a single primer extension based assay (ASSAY), that allows the identification of fusion transcripts without the need to know the fusion partner. A list of the fusion genes analyzed is given below.

Investigated fusion genes in alphabetical order			
Gene A	Gene B	FGFR2 all exons	Gene Z
