# Supplementary material for: Genomic architecture of FGFR2 fusions in cholangiocarcinoma and its implication for molecular testing
Source: Br J Cancer. 2022 Jul 23;127(8):1540–9. doi: 10.1038/s41416-022-01908-1 (PMC9553883; doi:10.1038/s41416-022-01908-1)
Supplement: Supplementary file 4 — DNA-Report [file 41416_2022_1908_MOESM4_ESM.pdf]

**Material:**	Block 12345/6789
**Clinical Details:**	Request for DNA based NGS FGFR2 fusion analysis
**Histopathology:**	Cholangiocarcinoma

Report:

Identified Fusion

5‘ Translocation Partner	3‘Translocation Partner	Translocation Supporting Reads	Ratio Translocation Reads
FGFR2 intron 17<br>*Break point:* chr10:123243212<br>GRCh37 - hg19	**BICC1 intron 2**<br>Break point: chr10:60461834<br>GRCh37 - hg19	**4321**	**49 %**

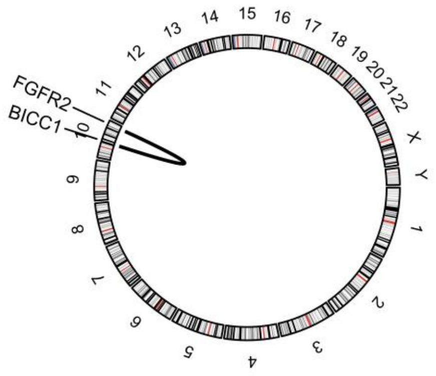

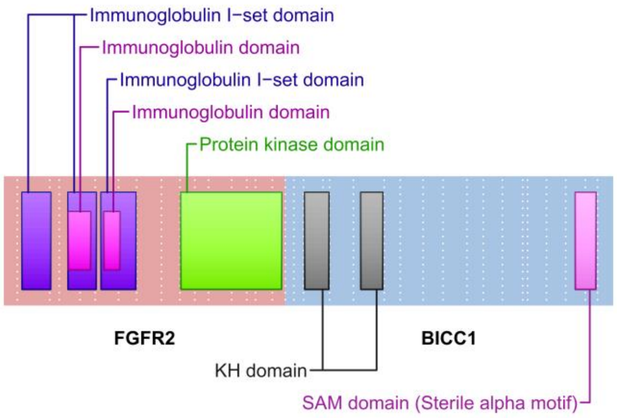

Comments – Interpretation:

In the present material, an FGFR2::BICC1 translocation with breakpoints in FGFR2 intron 17 and BICC1 intron 2 was detected at the DNA level, consistent in principle with a fusion of FGFR2 exon 17 (NM_000141.4) and BICC1 exon 3 (NM_001080512.1). The predicted fusion transcript results in the loss of the negative regulatory C-terminal region of FGFR2, while the kinase domain remains complete and intact. Further, a respective fusion protein would contain domains facilitating polymerization (KH and SAM) contributed by BICC1. The scientific literature describes both aspects as recurrent oncogenic mechanisms in cholangiocarcinoma (PMID: ######).

Additional information:

Pre-analytics

DNA input quantity:	80 ng	*Mapped Fragments:*	2000000
Tumor cell content:	70 %	*Unique mapped Fragments:*	999999

Performed analytics

Fusion analysis was performed on the morphologically confirmed and enriched tumor tissue (70% tumor cell content) using next-generation sequencing technology (SEQUENCING SYSTEM) using a hybrid capture based assay (ASSAY), that allows the identification of fusion transcripts without the need to know the fusion partner. A list of the fusion genes analyzed is given below.

Investigated fusion genes in alphabetical order			
Gene A	Gene B	FGFR2 intron 17, exon 17-18	Gene Z
